# Editorial: Community series in autoantibodies, volume II

**DOI:** 10.3389/fimmu.2025.1644057

**Published:** 2025-07-02

**Authors:** Aikaterini Kyriakou, Aikaterini Patsatsi

**Affiliations:** 2^nd^ Dermatology Department, School of Medicine, Aristotle University of Thessaloniki, Thessaloniki, Greece

**Keywords:** pemphigus, hidradenitis suppurativa (HS), bullous pemhigoid, bullous disease, epidermolysis bullosa

In volume II of the Research Topic on “Autoantibodies”, the impact of autoantibodies in patients with autoimmune skin diseases is further discussed.

Autoimmune bullous diseases (AIBDs) are a group of rare, chronic disorders characterized by the formation of bullae and erosions on the skin and/or mucous membranes. They result from an immune system attack on structural components of the skin that maintain cell-to-cell or cell-to-basement membrane adhesion. Pemphigus vulgaris, bullous pemphigoid and epidermolysis bullosa acquisita are some of the main representatives of AIBDs.

Previously, Eming et al. introduced a novel desmoglein 3 (Dsg3) EC5-binding antibody, termed 2G4, which may serve as a superior tool for various analyses related to PV. The aim of their recent study was to establish and validate standardized procedures to produce 2G4 IgG, enabling its use as a consistent and reliable tool in pemphigus research. This approach aims to facilitate the generation of comparable, high-quality data across different laboratory settings and time points. A comprehensive, point-by-point, quality-controlled IgG production protocol was established by Eming et al. that may serve as the foundation for standardized antibody assessment in PV research.

Previous case-control studies have suggested that environmental factors, such as exposure to pesticides and organic materials, dietary habits, and medication use, may play a significant role in the pathogenesis of PV. However, these studies were limited by the absence of geographically matched population controls and included fewer than three controls per case, reducing their statistical power and generalizability. Stone et al. conducted a case-control study to identify environmental and occupational risk factors contributing to the development of PV and bullous pemphigoid. Dietary factors containing thiol groups, such as leeks, tomatoes, and mustard oil, have been proposed as potential triggers for PV. Additionally, elevated levels of mental stress, the use of supplementary medications (e.g., calcium and multivitamins), and exposure to chemical cleaning products containing lime may be associated with an increased risk of developing both PV and bullous pemphigoid. These findings highlight the importance of incorporating lifestyle modifications into the routine management of patients with autoimmune blistering diseases.

PV is a potentially life-threatening autoimmune blistering disease characterized by the presence of autoantibodies targeting the desmosomal proteins named desmoglein (Dsg) 3 and Dsg1. Management of PV remains challenging and typically requires long-term administration of systemic corticosteroids in combination with additional immunosuppressive agents. In recent years, autoantibody-depleting therapies, such as rituximab, high-dose intravenous immunoglobulins (IVIG), and immunoadsorption, have emerged as effective treatment options. Enhancing the specificity of immunoadsorption for pathogenic autoantibodies could further improve the therapeutic efficacy and safety profile. Hofrichter et al. evaluated the ability of the newly developed prototypic Dsg1- and Dsg3-specific adsorbers to selectively remove circulating pathogenic autoantibodies from sera of PV patients. Their data clearly demonstrate that anti-Dsg3-specific IgG alone is sufficient to induce pathogenic effects both *in vitro* and *in vivo*. In contrast, depletion of Dsg3/1-specific autoantibodies results in a complete loss of pathogenicity. These findings support the concept that Dsg-specific immunoadsorption may represent a promising therapeutic strategy for the efficient removal of pathogenic autoantibodies in patients with severe PV.

In preclinical models of epidermolysis bullosa acquisita, Gross et al. previously demonstrated that depletion of regulatory T cells (Tregs) exacerbates autoantibody-induced, neutrophil-mediated skin inflammation and blistering. This heightened disease severity in Treg-depleted mice was associated with elevated cutaneous expression of interferon-gamma (IFN-γ). Given the association between Tregs depletion and increased cutaneous IFN-γ expression in EBA, Gross et al. sought to assess the therapeutic potential of IFN-γ inhibition in this disease. Specifically, they investigated whether targeting IFN-γ could modulate skin inflammation in a preclinical EBA model based on the passive transfer of type VII collagen (COL7)-specific antibodies into mice. Aside from a reduction in serum CXCL1 levels, a chemokine known to promote skin inflammation in EBA, cytokine expression in both serum and skin remained largely unchanged following IFN-γ blockade. These findings support the role of IFN-γ as a potential therapeutic target in EBA and may have broader implications for other autoimmune blistering diseases with similar pathogenic mechanisms, such as bullous pemphigoid and mucous membrane pemphigoid.

Hidradenitis suppurativa (HS) is a chronic inflammatory skin disease with both cutaneous and systemic manifestations. Emerging evidence suggests that immunoglobulins contribute to the dysregulated immune response observed in severe forms of HS. Recognizing these immunologic alterations is crucial, as patients may exhibit laboratory abnormalities indicative of chronic inflammation and immune activation. Misattributing these findings to unrelated disease processes may lead to unnecessary diagnostic procedures or inappropriate treatments, potentially resulting in harm to patients. Gauger et al. reported a case of a 23-year-old woman with Hurley stage III HS who was hospitalized and found to have persistent immunoglobulin-G4 (IgG4) elevation. Elevated IgG4 levels are often observed in chronic inflammatory conditions and may represent a compensatory immunomodulatory response. Although no previous reports have specifically described an association between hidradenitis suppurativa (HS) and IgG4 elevation, total IgG levels have been correlated with HS disease severity. Gauger et al. suggest that IgG4 may hold potential as a biomarker for disease monitoring in HS. Clinicians should be aware of this possible association to avoid misinterpretation of laboratory results and to better guide patient management.

